# Prevalence and multilevel factors associated with clinical breast examination uptake among women in sub-Saharan Africa: a systematic review and meta-analysis

**DOI:** 10.3389/fonc.2026.1856988

**Published:** 2026-07-31

**Authors:** Silas Selorm Daniels-Donkor, Amanda Datesman, Ha Do Byon, Donald Babamomba Amenah, Basiru Dawda, Molly J. Yeo, Emma M. Mitchell

**Affiliations:** 1 Department of Nursing Research, University of Virginia School of Nursing, Charlottesville, VA, United States; 2 Department of Family and Community Health, University of Maryland School of Nursing, Baltimore, MD, United States

**Keywords:** breast cancer screening, clinical breast examination, meta-analysis, multilevel factors, sub-Saharan Africa, systematic review

## Abstract

**Background:**

Clinical breast examination (CBE) is a feasible early-detection method in low-resource settings, but critical gaps remain regarding its uptake in sub-Saharan Africa. We estimated the prevalence of CBE uptake and synthesized multilevel factors associated with uptake, organized within a socio-ecological framework.

**Methods:**

We conducted a PRISMA-guided systematic review and meta-analysis of cross-sectional studies assessing self-reported CBE uptake. PubMed, Embase, Web of Science, CINAHL, Africa-Wide Information, Google Scholar, and gray literature were searched. Random-effects meta-analysis was used to pool prevalence estimates and adjusted odds ratios for determinants.

**Results:**

The pooled prevalence of CBE uptake was 13% (95% CI, 9-18%). Pooled determinant analyses showed that higher educational attainment, greater household wealth, health insurance coverage, and recent health-facility contact were associated with higher CBE uptake. Internet use, radio listening, and rural residence were not statistically significant in the pooled analysis. Additional non-pooled findings suggested that age, breast cancer awareness, breast self-examination, reproductive and maternal health-service contact, and empowerment-related factors are also associated with CBE uptake.

**Conclusion:**

CBE uptake in sub-Saharan Africa is low and socioecologically patterned, underscoring the need for integrated, equity-focused early detection strategies at the individual, community, and health-system levels.

## Introduction

Breast cancer is the leading cause of cancer death among women in sub-Saharan Africa (SSA), claiming over 68,000 lives in 2022 alone, many of which could have been prevented through early detection (1–3). The five-year survival rate in numerous SSA contexts remains significantly low, approximately 40% in South Africa compared to over 80% in high-income nations (4), indicating that most cancers are detected late. The World Health Organization’s Global Breast Cancer Initiative accordingly aims to reduce breast cancer mortality by 2.5% per year by 2040 through enhanced screening and treatment (2). However, breast cancer screening in SSA remains inadequate, undermining early detection and timely diagnosis (2, 5).

In high-income countries, widespread mammographic screening has improved survival rates (2, 6). However, due to cost, infrastructure, and workforce limitations, mammography programs frequently prove impractical in low-resource settings (2, 7). The World Health Organization (WHO) recommends clinical breast examination (CBE) as a feasible early-detection method in these contexts, due to its affordability and relative simplicity of implementation (8, 9). CBE involves a qualified healthcare professional performing a physical assessment of the breasts, which can facilitate early cancer detection when performed regularly (10).

Notwithstanding the WHO’s approval of CBE as a feasible early detection method in low-resource settings, more than 85% of women in SSA remain unscreened (11). For example, pooled Demographic and Health Survey (DHS) data from seven African countries (Burkina Faso, Côte d’Ivoire, Ghana, Kenya, Mozambique, Senegal, Tanzania) show that only about 19.2% of women report ever having had a CBE (12). A separate population-based study conducted in four SSA countries reported a comparable low prevalence of 12.9% for having undergone CBE (13). This suggests that most women in these settings, particularly those without symptoms, have never undergone CBE, which may contribute to delayed detection (14).

Screening coverage is highly inequitable in SSA (15). Women of higher socioeconomic status are far more likely to be screened (15). Studies consistently find that belonging to a higher wealth index is associated with greater CBE uptake (13). For example, studies consistently show higher CBE uptake among women in wealthier households, with the richest women often having 2 times the odds of screening compared to the poorest (11, 16, 17). Notably, women’s contact with the health system is a strong correlate of CBE uptake (11, 18, 19). For instance, in Lesotho, women who had visited a health facility in the past year were about 1.6 times more likely to have had a CBE than those who had not (18).

While individual studies have identified factors associated with CBE uptake, critical gaps remain in understanding the multilevel factors associated with CBE uptake across SSA. We lack contemporary evidence that systematically synthesizes CBE prevalence across diverse settings and regions and integrates findings on factors associated with CBE at the individual, community, and health-system levels to guide equitable early detection strategies. Our study directly addresses this gap. Using a systematic review and meta-analysis, we synthesize available evidence to estimate pooled CBE uptake in SSA and summarize factors associated with CBE uptake using a social-ecological model (SEM) framework (20), examining influences from the individual level through community and health-system factors. The SEM presents an ecological framework for health promotion that emphasizes different levels of influence on health behavior, including individual, interpersonal, organizational, community, and public policy factors (20). Health behavior is often influenced by the interplay between individuals and their social and physical environments (21). This evidence is needed to inform targeted, feasible interventions and advance the goals of the WHO Global Breast Cancer Initiative.

## Methods

A systematic review of the literature on the prevalence and multilevel factors associated with CBE uptake among women in SSA was conducted in accordance with the Preferred Reporting Items for Systematic Reviews and Meta-Analyses (PRISMA) reporting guidelines (22). The protocol for this review was registered with the International Prospective Register of Systematic Reviews (PROSPERO) before the comprehensive literature search, data extraction, and analysis (CRD420251109189). The review question was defined using PICOS: population, women residing in SSA; intervention/exposure, CBE uptake and associated multilevel factors; comparators, reference groups used in the included studies where applicable; outcomes, prevalence of CBE uptake and adjusted odds ratios for factors associated with uptake; and study design, cross-sectional studies.

### Search strategy

A database search strategy was developed and conducted by a medical librarian for this review. An initial PubMed search identified relevant citations to inform the search strategy. The titles and abstracts of the relevant articles were examined for additional keywords and index terms to inform development of a comprehensive search strategy, which is provided in **Supplementary Table 2**. PubMed, CINAHL (EBSCO), Africa-Wide Information (EBSCO), Embase (Elsevier), and select indexes of Web of Science (as noted in the search appendix) were searched. Africa-Wide Information is a multidisciplinary database on the EBSCO platform that indexes health and social science research with a focus on African contexts. The search was also adapted for use in Google Scholar and limited to the first 1000 relevant records. In addition to bibliographic database searches, we conducted gray literature searches to identify government publications, policy documents, national cancer control strategies, screening guidelines, and programmatic sources on CBE uptake and determinants in SSA. Sources searched included the World Health Organization Institutional Repository for Information Sharing (WHO IRIS), the World Health Organization Regional Office for Africa (WHO AFRO), the World Health Organization Global Health Observatory (WHO GHO), World Health Organization Global Breast Cancer Initiative (WHO GBCI) materials, African Index Medicus (AIM), the International Agency for Research on Cancer/Global Cancer Observatory (IARC/GCO), and national Ministry of Health or national cancer-control sources (**Supplementary Table 2** and **3**). Results across all databases were limited to English-language articles, as translation services outside the review team were not available. Only articles published between 1/1/2010 and 12/23/2025 were retrieved in the search. This time period was chosen to capture current evidence after WHO guidance identified CBE as a feasible early-detection approach in low-resource settings, and to align the review with subsequent WHO guidance emphasizing early diagnosis and comprehensive breast cancer management (23, 24).

### Inclusion and exclusion criteria

Studies were eligible for inclusion if they were published in English and reported CBE uptake among women residing in SSA. Specific inclusion criteria were as follows: (1) If the respondents were women residing in SSA (2) if the outcome focused on CBE uptake, defined as self-reported history of having undergone CBE performed by a qualified health professional, as defined by each primary study (typically ever having had CBE); (3) if the study outcomes examined prevalence of CBE uptake and/or factors associated with CBE uptake; (4) if the study design was cross-sectional studies; and (5) if the articles published in English. Studies were excluded if they did not report CBE findings separately and instead combined CBE findings with breast self-examination (BSE) or mammography.

### Study selection

Two independent authors (SSD-D, DA) screened articles by title and abstract in Covidence.

The same reviewers then examined full-text reports for study eligibility. Discrepancies were resolved through consensus.

### Data extraction

Three authors (DA, BD, MY) extracted the study characteristics onto the data extraction sheet. From each study, the following information was extracted: the authors, year of publication, country, sample size, study design, prevalence rates, and factors associated with CBE. The information extracted from the various studies was cross-checked by another reviewer (SSD-D) for accuracy.

### Quality assessment of included articles

All the eligible articles were assessed for quality by two independent authors using the Joanna Briggs Institute (JBI) Critical Appraisal Checklist for Cross-Sectional Studies (25). All discrepancies were resolved through consensus. Studies were categorized as high quality if ≥6 checklist items were rated ‘Yes’. No studies were excluded, as none received fewer than 6 scores on their respective checklists. (**Table 1**).

**Table 1:** Critical appraisal of included cross-sectional studies using the JBI checklist.

Study	Q1	Q2	Q3	Q4	Q5	Q6	Q7	Q8	Total yes	Overall appraisal
Abubakari et al. (45),									8	High
Abuhay et al. (34),							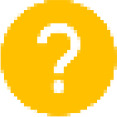		7	High
Addo et al. (15),							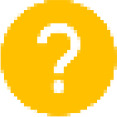		7	High
Afaya et al. (18),							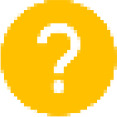		7	High
Ali, 2025 (40)							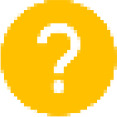		7	High
Antabe et al. (33),							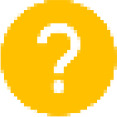		7	High
Ayebeng et al. (12),							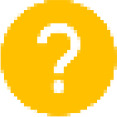		7	High
Ba et al. (13),							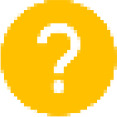		7	High
Bamusi et al. (3),							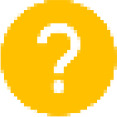		7	High
Bawuah et al (35),							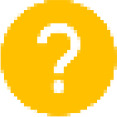		7	High
Daniels-Donkor et al (44),							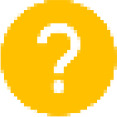		7	High
Gebreegziabher et al. (17),							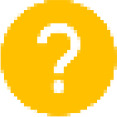		7	High
Hailegebireal et al. (11),							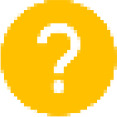		7	High
Kangmennaang et al. (32),							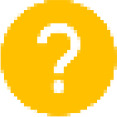		7	High
Okyere et al. (43),							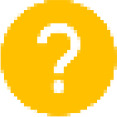		7	High
Okyere et al. (41),							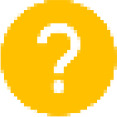		7	High
Saaka et al. (42),							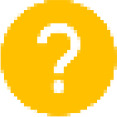		7	High
Lemma Seifu et al. (16),							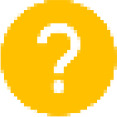		7	High
Tibenderana et al. (19),							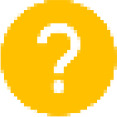		7	High


 = Yes; 
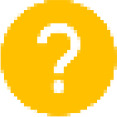
 = Unclear.

Questions

1. Were the criteria for inclusion in the sample clearly defined?

2. Were the study subjects and the setting described in detail?

3. Was the exposure measured in a valid and reliable way?

4. Were objective, standard criteria used for measurement of the condition?

5. Were confounding factors identified?

6. Were strategies to deal with confounding factors stated?

7. Were the outcomes measured in a valid and reliable way?

8. Was appropriate statistical analysis used?

### Statistical analysis

All quantitative syntheses were performed using random-effects meta-analysis in R, primarily using the meta package (26). To calculate the pooled prevalence of CBE uptake, study-specific prevalence proportions were synthesized on the logit scale using a random-effects model with REML estimation of between-study variance (27). Random-effects models were selected *a priori* because true effects were predicted to vary among countries, populations, and health-care systems. Logit-scale pooling was used to stabilize variances and improve confidence-interval performance when proportions are small (28). The pooled estimates were then back-transformed and reported as proportions with 95% confidence intervals (29). The I² statistic was used to assess between-study heterogeneity (30). The same random-effects framework was used for the regional subgroup analyses (East Africa, West Africa, and Southern Africa). For factors associated with CBE uptake, this meta-analysis pooled adjusted odds ratios. For each factor associated with CBE, we conducted a random-effects meta-analysis only when at least three studies reported adjusted odds ratios with 95% confidence intervals (31). To avoid redundant weighting from overlapping DHS datasets, the prevalence meta-analysis included only one country-specific estimate per unique country-survey wave (**Supplementary Table 1**). Leave-one-out sensitivity analysis was conducted by sequentially omitting one country’s survey prevalence estimate at a time and re-estimating the pooled prevalence using the same random-effects logit-transformed proportion model. For determinant meta-analyses, only one adjusted estimate per unique country-survey wave was retained for each factor; overlapping estimates from duplicate country-specific analyses or multi-country DHS analyses were summarized narratively rather than pooled (**Supplementary Table 1**). This approach ensured that each underlying country-survey wave contributed only once to each pooled estimate, avoiding multiple counting of the same DHS respondents. The reported adjusted ORs and 95% confidence intervals from the individual studies were extracted and then converted to log-transformed odds ratios, and their standard errors were calculated from reported adjusted odds ratios and 95% confidence intervals, and the odds ratios were pooled using a random-effects inverse-variance model with REML estimation of τ² and Hartung–Knapp-adjusted confidence intervals. We reported 95% prediction intervals to reflect the expected range of effects in comparable future settings. Heterogeneity was assessed using the I² statistic, with statistical significance established at α = 0.05.

## Results

**Figure 1:**
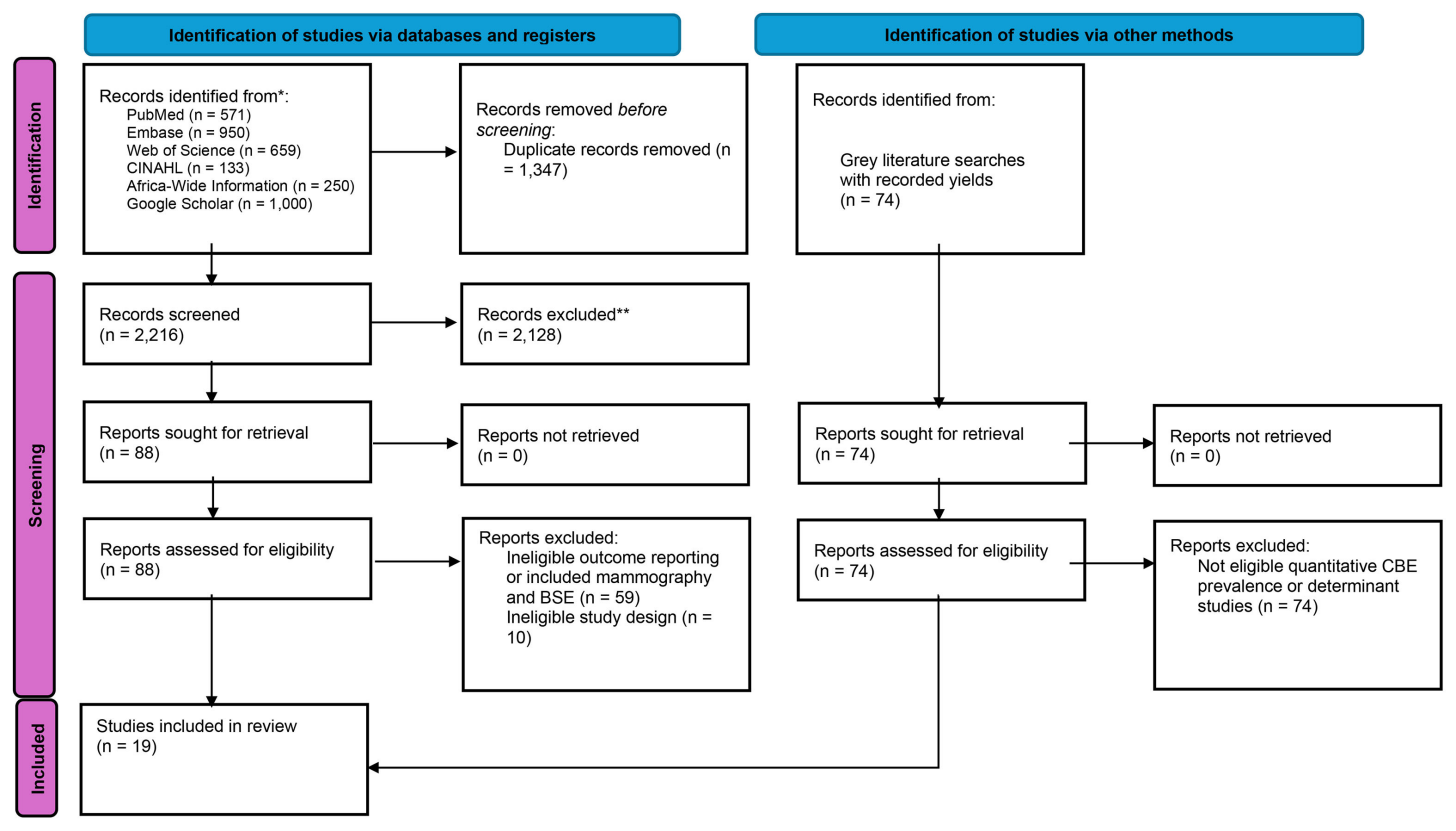
PRISMA flow diagram.

A systematic search of six databases (PubMed, Embase, Web of Science, Google Scholar, Africa-Wide Information, and CINAHL) yielded 3,563 records. After removing 1,347 duplicates, 2,216 records were screened, and 2,128 were excluded based on title and abstract review. The remaining 88 studies were assessed for full-text eligibility. Of these, 69 full-text articles were excluded for ineligible outcome reporting (n = 59) or ineligible study design (n = 10). Nineteen studies met the eligibility criteria and were included in this review (**Figure 1**). The gray literature search did not identify any additional eligible studies. The included studies represented women from three SSA: West Africa (Ghana, Senegal, Côte d’Ivoire, Burkina Faso), East Africa (Kenya, Tanzania), and Southern Africa (Mozambique, Lesotho, Namibia). The characteristics of the included studies, including author, year, country, sample size, study design, and factors associated with CBE, are summarized in **Table 2**.

**Table 2:** Study characteristics of the included articles.

Author(s), year	Country/region	Data source/setting	Design	Study population	Sample size (N)	Key factors associated with CBE uptake
Abebe Gebreegziabher et al. (2024) (17)	Ghana	Ghana Demographic and Health Survey (GDHS)	Cross-sectional (secondary DHS data)	Women 15–49 years	15,013 (weighted)	Age, education, wealth index, contraception use, health insurance, media exposure (radio, newspaper), facility visit in the past 12 months, and distance to health facility.
Abubakari et al. (2025) (45)	Ghana (Kumasi, Ashanti)	Community-based survey (Aboabo & Asawase communities)	Cross-sectional	Islamic women (≥18 years)	500	Islamic norms/religiosity, knowledge, perceived benefits, health service availability
Abuhay et al. (2025) (34)	Kenya	Kenya Demographic and Health Survey (KDHS)	Cross-sectional (secondary DHS data)	Women, 15–49 years	16,649 (weighted)	Age, education, parity, media exposure, religion, residence, wealth index.
Addo et al. (2023) (15)	Namibia; Burkina Faso; Côte d’Ivoire; Kenya; Lesotho	Demographic and Health Surveys from 5 countries	Cross-sectional (pooled DHS)	Women, 15–49 years	45,945 (weighted)	Distance to facility, age, residence, education, media exposure(newspaper, radio), wealth, insurance, marital status
Afaya et al. (2023) (18)	Lesotho	2014 Lesotho Demographic and Health Survey(LDHS),	Cross-sectional (secondary DHS data)	Women, 15–49 years	6,584 (weighted)	Health insurance, current pregnancy, media use (newspaper/magazine), breast cancer awareness, recent breast self-exam, recent facility visit
Ali (2025) (40)	Kenya	Kenya Demographic and Health Survey (KDHS)	Cross-sectional (secondary DHS data)	Women, 15–49 years	10,267 (weighted)	Age, employment, marital status, wealth index, education
Saaka et al. (2025) (42)	Ghana	Ghana Demographic and Health Survey (GDHS),	Cross-sectional (secondary DHS data)	Women 15–49 years	15,014 (weighted)	Women’s decision-making autonomy, employed, health insurance, facility visit in the past 12 months, listened to radio, education, age, employment, health insurance, residence, wealth, religion, media exposure (radio),
Antabe et al. (2020) (33)	Kenya	Kenya Demographic and Health Survey (KDHS)	Cross-sectional (secondary DHS data)	Women 15–49 years	~14,734	Education, marital status, province/region, residence (rural), employment, wealth quintile, health insurance(no), age
Ayebeng et al. (2025) (12)	Burkina Faso; Côte d’Ivoire; Ghana; Kenya; Mozambique; Senegal; Tanzania	Demographic and Health Surveys from 7 countries	Cross-sectional (pooled DHS)	Women, 15–49 years	65,486 (weighted)	Access barriers (permission, distance, cost, traveling alone)
Ba et al. (2020) (13)	Burkina Faso; Côte d’Ivoire; Kenya; Namibia	Demographic and Health Surveys from 4 countries	Cross-sectional (pooled DHS)	Women, 15–49 years	39,646 (weighted)	Education, age, health insurance coverage, household wealth index
Bamusi et al. (2024) (3)	Tanzania	2022 Tanzania Demographic and Health Survey (TDHS)	Cross-sectional (secondary DHS data)	Women 20–49 years	4,216 (weighted)	Age, education
Bawuah & Edefo (2025) (35)	Senegal	2023 Senegal Demographic and Health Survey (SDHS)	Cross-sectional	Women, 15–49 years	4,532 (weighted)	Wealth index, employment, radio exposure, age, distance to a health facility.
Daniels-Donkor & Afaya (2025) (44)	Kenya	2022 Kenya Demographic and Health Survey (KDHS)	Cross-sectional (secondary DHS data)	Women, 15–49 years	16,649 (weighted)	frequency of internet use in the last month, age, marital status, residence, education, media exposure, facility visit in last 12 months, and wealth index.
Hailegebireal et al. (2024) (11)	Burkina Faso, Côte d’Ivoire, Lesotho, Kenya, Namibia, Tanzania	Demographic and Health Survey from 6 countries.	Cross-sectional (pooled DHS)	Women, 15–49 years	95,248 (weighted)	Age, education, cohabitation status, household wealth, urban residence, parity, recent health facility visit, newspaper reading
Kangmennaang et al. (2019) (32)	Namibia	Namibia Demographic and Health Survey (NDHS)	Cross-sectional	Women 15–49 years	9,176 (weighted)	Age, education, marital status, health insurance, breast self-exam, contact with health professionals in 12 months, religion, place of residence, employment, parity
Okyere et al. (2024) (43)	Cote d’Ivoire	Cote d’Ivoire Demographic and Health Survey(IDHS)	Cross-sectional (secondary DHS data)	Women 15–49 years	14,685 (weighted)	Age at menarche (primary exposure), age, education, residence, media use (newspaper), wealth index,
Okyere et al. (2024) (41)	Ghana	2022 Ghana Demographic and Health Survey (GDHS)	Cross-sectional (secondary DHS data)	Women, 15–49 years	15,013 (weighted)	Internet use frequency, age, education, marital status, wealth index, residence, media exposure, distance to facility.
Lemma Seifu et al. (2024) (16)	Burkina Faso, Côte d’Ivoire, Lesotho, Kenya, Namibia, Tanzania	Demographic and Health Surveys from 6 countries	Cross-sectional (pooled DHS)	Women, 15–49 years	80,058 (weighted)	Age, education, wealth, media exposure, contraceptive use, facility visit in the past 12 months, pregnancy status
Tibenderana et al. (2024) (19)	Tanzania	2022 Tanzania Demographic and Health Survey (TDHS),	Cross-sectional (secondary DHS data)	Women, 15–49 years	15,189 (weighted)	Age, education, facility visit in the past 12 months, insurance, wealth index, residence.

### Prevalence of clinical breast examination

Prevalence data, including reported prevalence rate and its confidence interval on CBE among women, were available from 12 country-specific estimates and included in the pooled-prevalence meta-analysis. The meta-analytic set for the pooled prevalence included population-based cross-sectional studies (3, 12, 15, 17, 18, 32–35) conducted across East, Southern, and West Africa, published from 2019 to 2025.

**Figure 2:**
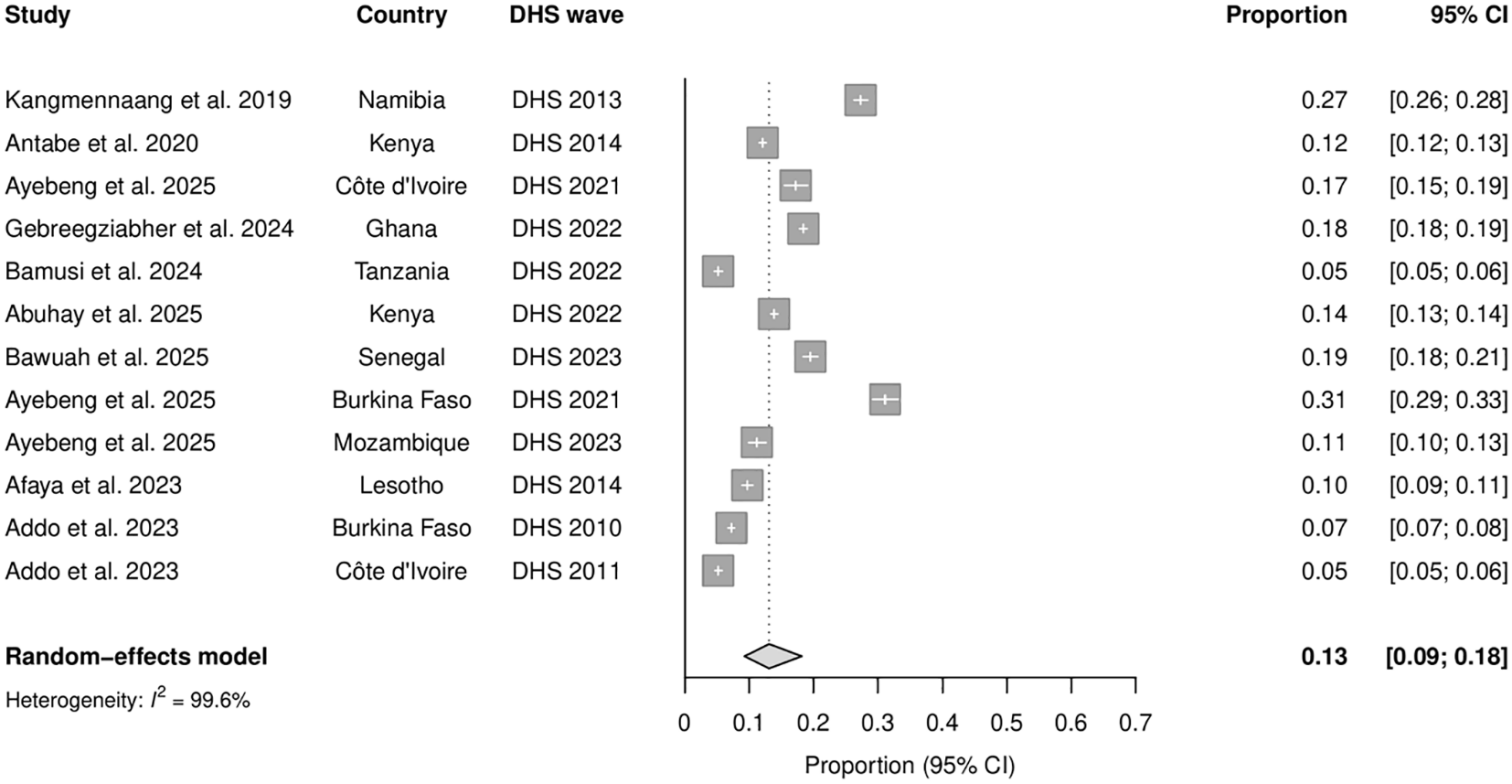
Forest plot of the pooled prevalence of CBE uptake.

**Figure 3:**
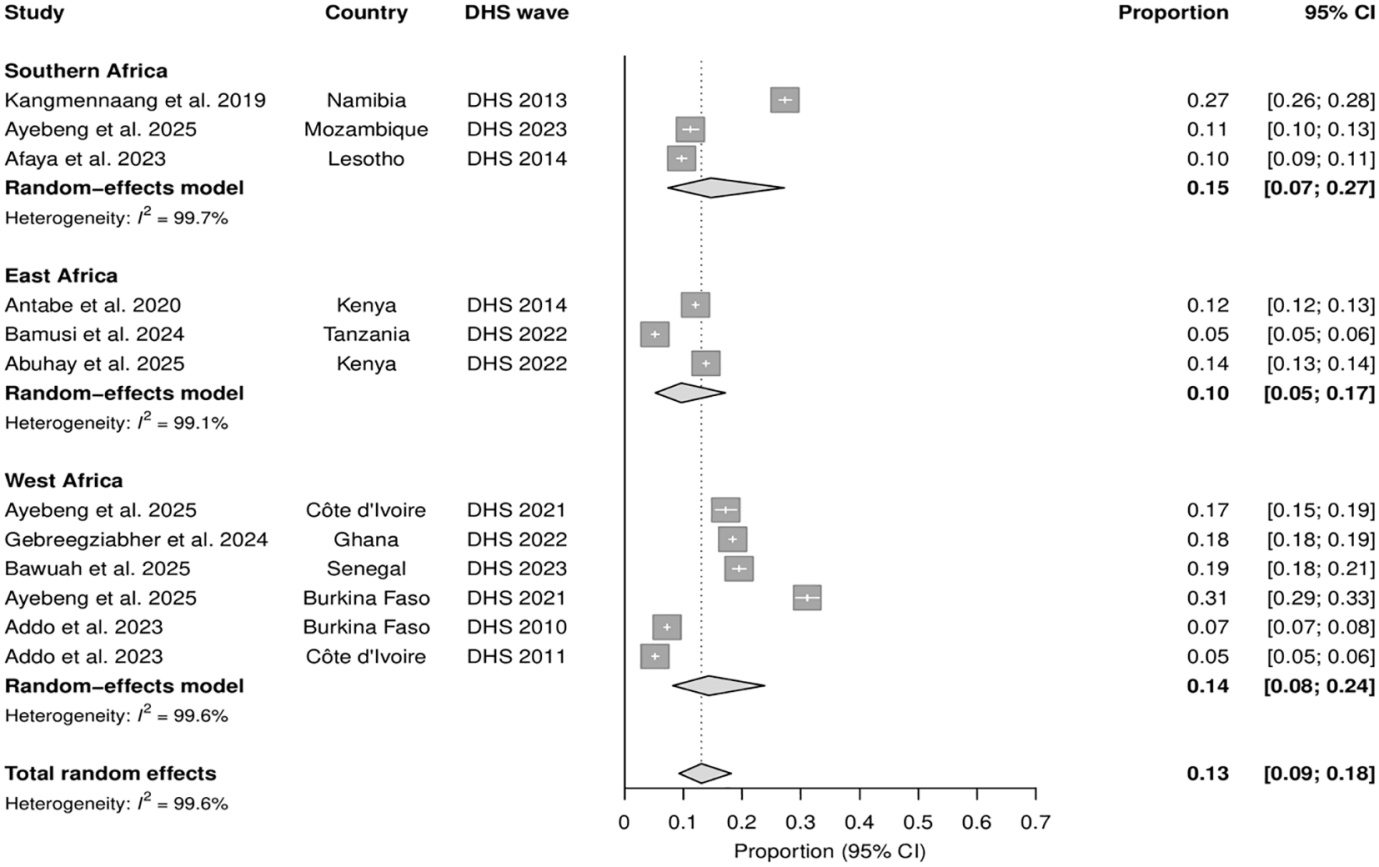
Forest plot of the pooled prevalence of CBE uptake by sub-region in SSA.

In the random-effects meta-analysis, the pooled prevalence of CBE uptake was 0.13 (95% CI, 0.09-0.18), corresponding to an estimated 13% of women having had CBE, with heterogeneity of 99.6% (**Figure 2**). Study-level prevalence estimates ranged from 0.05 (3, 15) to 0.31 (12). In a regional subgroup analysis, the pooled prevalence estimates were: East Africa (3, 33, 34): 0.10 (95% CI, 0.05–0.17; I² = 99.1%); Southern Africa (12, 18, 32): 0.15 (95% CI, 0.07-0.27; I² = 99.7%); West Africa (12, 15, 17, 35): 0.14 (95% CI, 0.08-0.24; I² = 99.6%). Heterogeneity remained high within each subgroup (I² >99%) (**Figure 3**), and the test for subgroup differences was not statistically significant (Q = 1.21, df = 2, p = 0.548), indicating no evidence of differential prevalence across regions (**Figure 3**). This meta-analysis synthesized proportions (prevalence estimates) without formally assessing publication bias using funnel plots or Egger’s regression test, as these methods are not usually recommended for meta-analyses of proportions and are challenging to interpret in instances of extreme heterogeneity (36). This is because publication bias tests were developed primarily for comparative effect estimates, whereas single-arm prevalence estimates lack a clear direction of effect (36). Sample–size–based funnel plots and Doi plots using the Luis Furuya-Kanamori (LFK) index have been proposed as alternative approaches to assess asymmetry or small-study effects in meta-analyses of proportions. However, these approaches do not fully resolve the interpretive challenges of single-arm prevalence meta-analysis and remain difficult to interpret when between-study heterogeneity is very high (36–38). Therefore, we did not apply these methods and instead sought to reduce reporting bias by conducting a comprehensive search of databases and gray literature. The leave-one-out sensitivity analysis revealed that the pooled prevalence estimate ranged from 12.0% to 14.2% when each country-survey estimate was sequentially removed, suggesting that no individual country-survey estimate significantly influenced the overall pooled prevalence. The persistently high heterogeneity indicates that the stability of the pooled estimates should not be interpreted as evidence of uniform CBE uptake across various settings.

### Factors associated with clinical breast examination uptake

**Figure 4:**
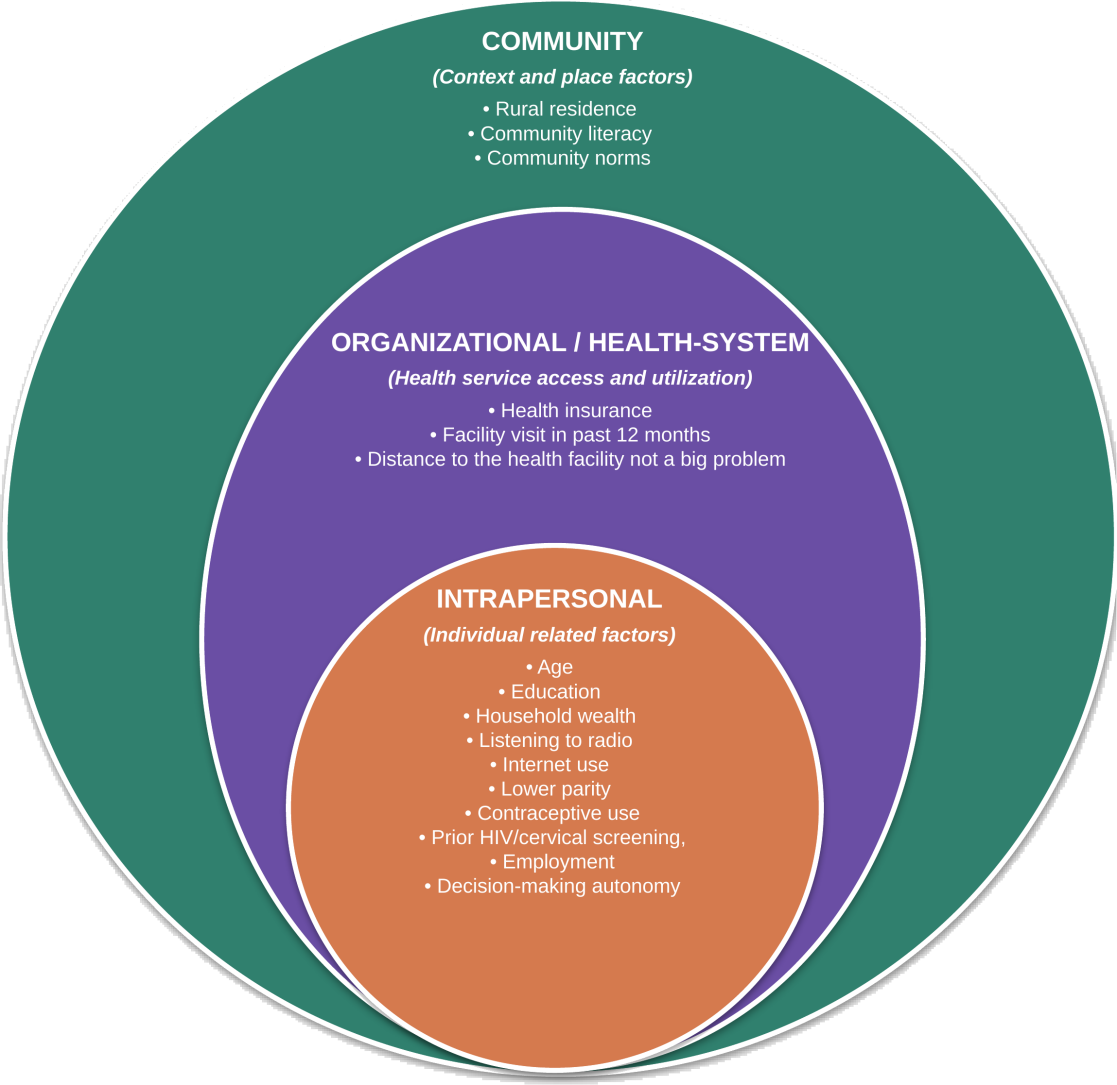
Modified socio-ecological model presenting the multilevel factors associated with CBE.

Factors associated with CBE uptake were organized using the socio-ecological model, separating individual-level, community-level, and health-system-level factors (39) (**Figure 4**). When at least three studies reported factors associated with CBE with adjusted ratios and 95% confidence intervals, we conducted a pooled random-effects meta-analysis and reported the pooled estimates, heterogeneity measures, and prediction intervals. Factors reported in only one study, or without adjusted odds ratios and confidence intervals, were summarized narratively. We did not conduct funnel plots and egger’s tests for determinant meta-analyses because no determinant contrast had 10 or more independent estimates.

### Individual-level factors

#### Educational attainment

**Figure 5:**
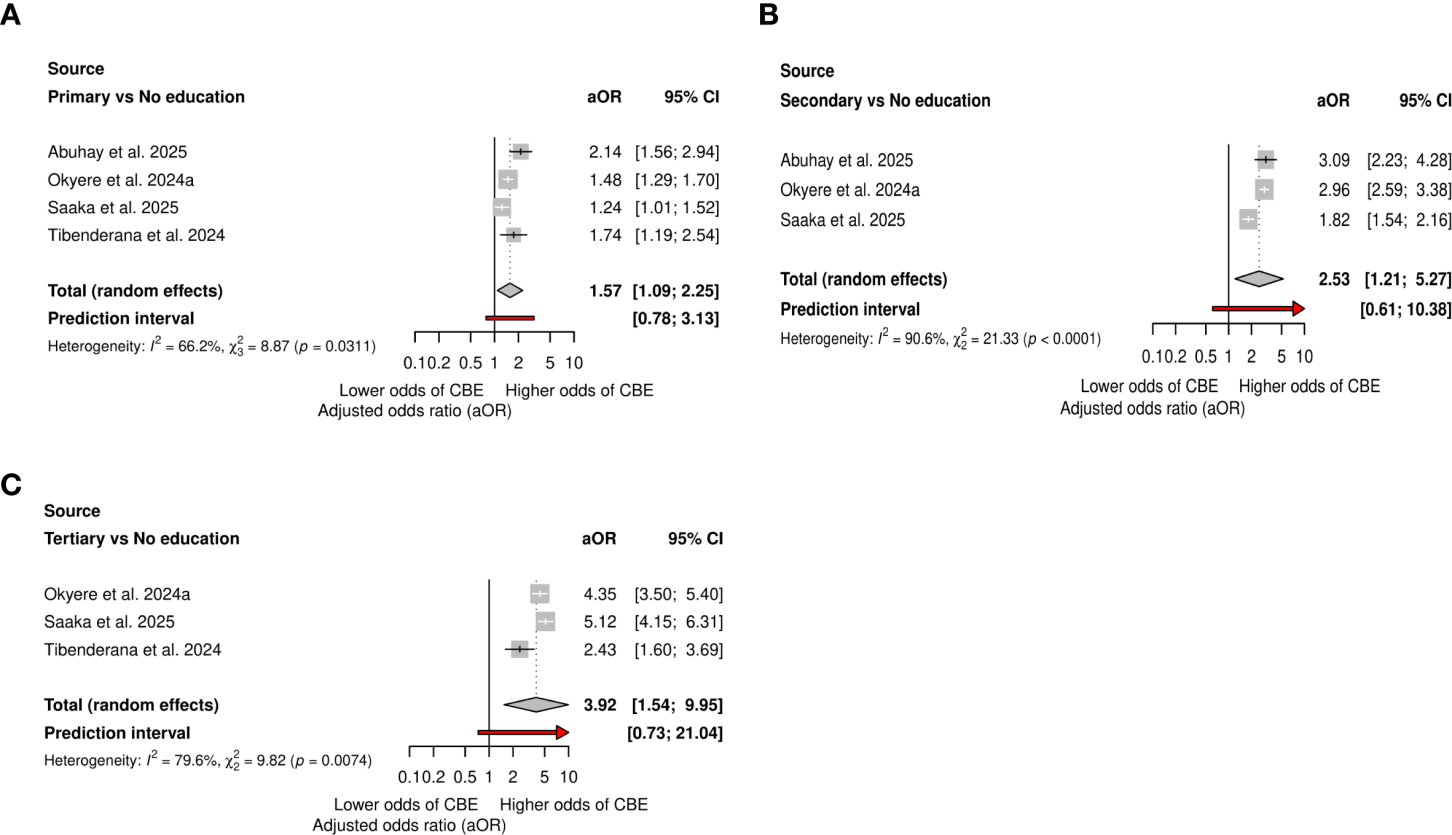
Forest plots of the association between educational attainment and clinical breast examination uptake: **(a)** primary education versus no education; **(b)** secondary education versus no education; and **(c)** tertiary education versus no education.

Studies (3, 11, 15–17, 19, 34, 40–42) found that educational attainment was associated with CBE. In the pooled analysis, higher educational attainment was associated with higher odds of CBE uptake (primary education: OR, 1.57; 95% CI, 1.09-2.25; secondary education: OR, 2.53; 95% CI, 1.21-5.27; tertiary education: OR, 3.92; 95% CI, 1.54-9.95) (**Table 3**) (**Figures 5a-c**).

**Table 3:** Pooled factors associated with CBE uptake organized by the socio-ecological model.

Factors	Studies (k)	Pooled OR (95% CI)	I² (%)	95% prediction interval
Individual-level factors				
Primary education vs no education	4	1.57 (1.09-2.25)	66.2	0.78-3.13
Secondary education vs no education	3	2.53 (1.21-5.27)	90.6	0.61-10.38
Tertiary education vs no education	3	3.92 (1.54-9.95)	79.6	0.73-21.04
Middle wealth vs poorest	3	1.44 (1.22-1.69)	0.0	1.08-1.92
Richer wealth vs poorest	3	1.64 (1.36-1.98)	0.0	1.20-2.25
Richest wealth vs poorest	3	2.21 (1.67-2.91)	0.0	1.56-3.12
Listening to radio (yes vs no)	3	1.27 (0.98-1.64)	43.6	0.82-1.95
Internet use (yes vs no)	3	1.08 (0.34-3.51)	90.6	0.12-10.09
Community-level factors				
Rural residence vs urban residence	4	0.82 (0.63-1.06)	70.3	0.52-1.29
Organizational and health-system factors				
Health insurance (yes vs no)	3	1.99 (1.03-3.81)	75.6	0.58-6.81
Facility visit in past 12 months (yes vs no)	3	1.40 (1.20-1.64)	0.0	1.16-1.69

OR, odds ratio; CI, confidence interval; PI, prediction interval; I², heterogeneity statistic; k, number of studies. All pooled estimates are from random-effects meta-analysis.

#### Household wealth index

**Figure 6:**
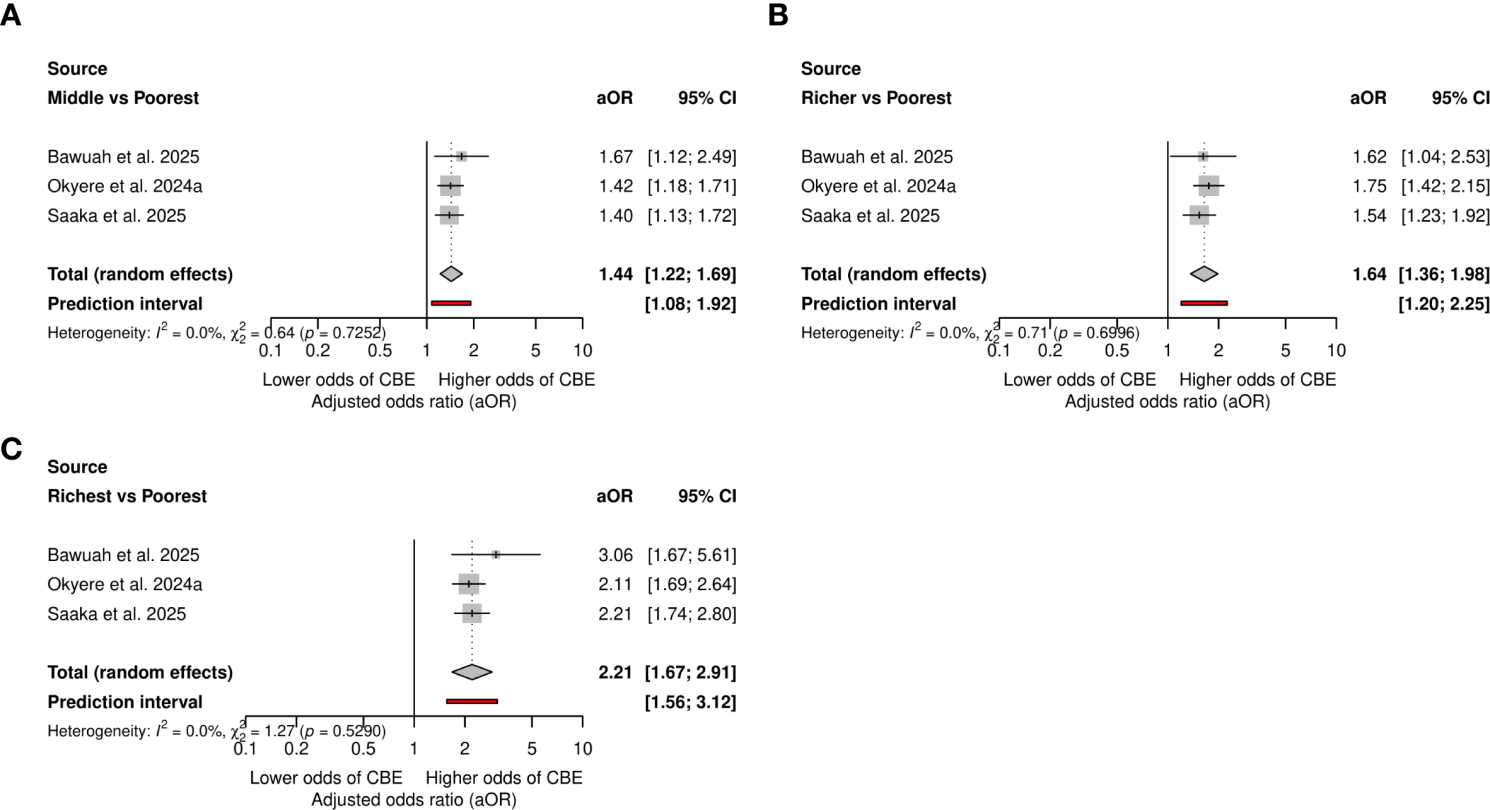
**(a)** Wealth index (middle vs poorest). **(b)** Wealth index (richer vs poorest). **(c)** Wealth index (richest vs poorest).

Studies (11, 12, 16, 17, 19, 34, 35, 41–43) found that household wealth index was associated with CBE uptake. In the pooled analysis, compared with women from the poorest wealth category, the odds were higher for middle (OR, 1.44; 95% CI, 1.22-1.69), richer (OR, 1.64; 95% CI, 1.36-1.98), and richest (OR, 2.21; 95% CI, 1.67-2.91) wealth categories (**Table 3**) (**Figures 6a-c**).

#### Internet usage

**Figure 7:**
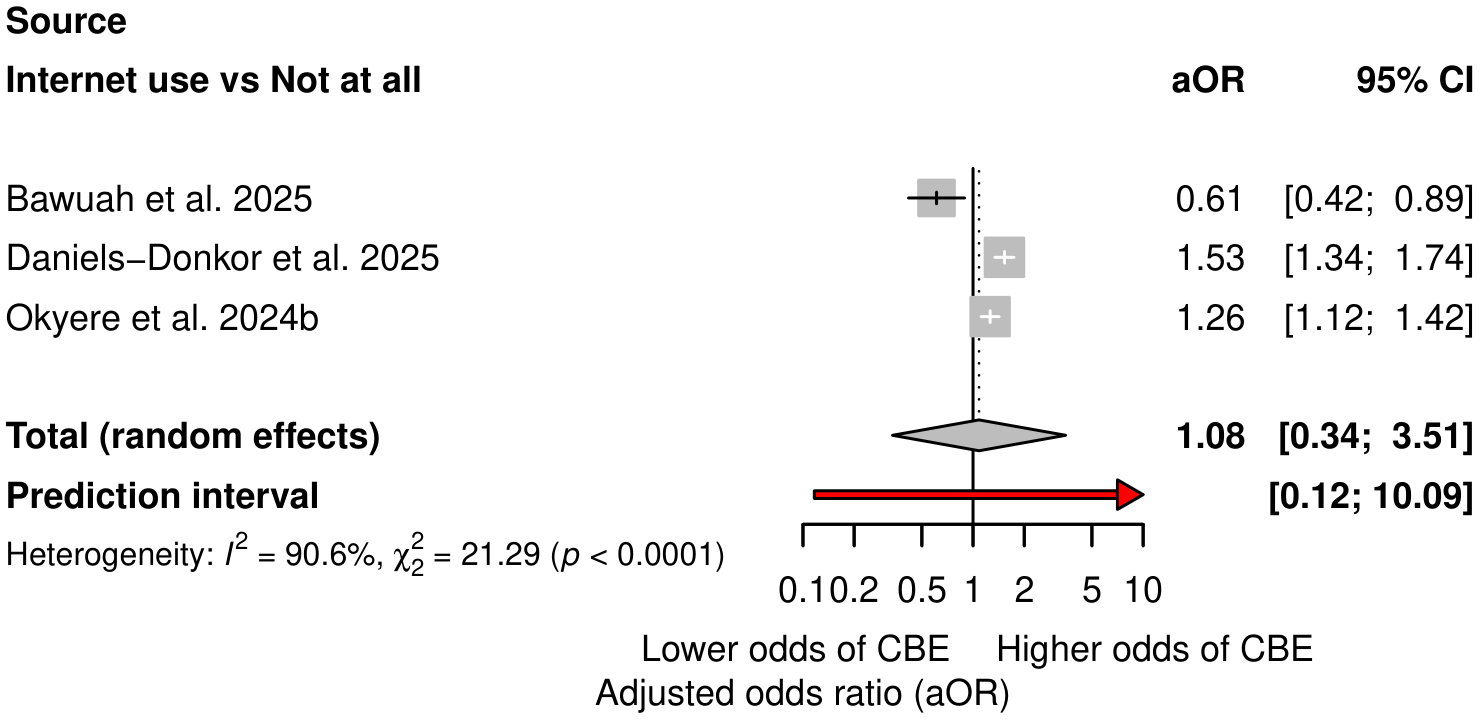
Internet use (yes vs no).

Three studies (35, 41, 44) found that internet use was associated with CBE. However, in the pooled analysis, internet use was not statistically associated with CBE (OR, 1.08; 95% CI, 0.34-3.51). The wide confidence and prediction intervals, together with high heterogeneity, indicate substantial variation across the included estimates (**Table 3**) (**Figure 7**).

#### Listening to the radio

**Figure 8:**
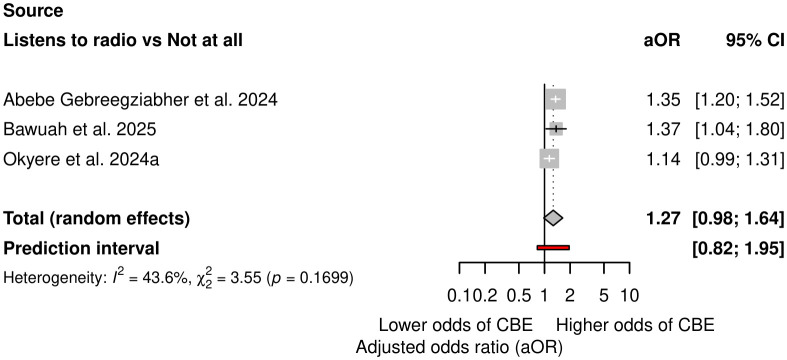
Listening to radio (yes vs no).

Studies (15, 17, 35, 42, 43) identified radio listening as associated with CBE uptake. In the pooled analysis, radio listening showed a positive but not statistically significant association with CBE uptake (OR, 1.27; 95% CI, 0.98-1.64) (**Table 3**) (**Figure 8**).

Other individual-level factors were identified in studies that could not be pooled in the meta-analysis due to overlapping data sources, fewer than 3 independent estimates, or non-comparable measures. Age was generally associated with CBE uptake across several studies (17, 19, 34, 40, 43). Others included breast cancer awareness (18), recent breast self-examination (18, 32), pregnancy status and parity (16, 18), modern contraceptive use (16, 17, 34), employment (35, 40, 42), decision-making autonomy (3, 42), and broader media exposure such as newspapers/magazines (18)or television (40). One study also reported that suicidal ideation was associated with lower odds of CBE uptake (35) (**Table 2**).

### Community-level factors

#### Place of residence

**Figure 9:**
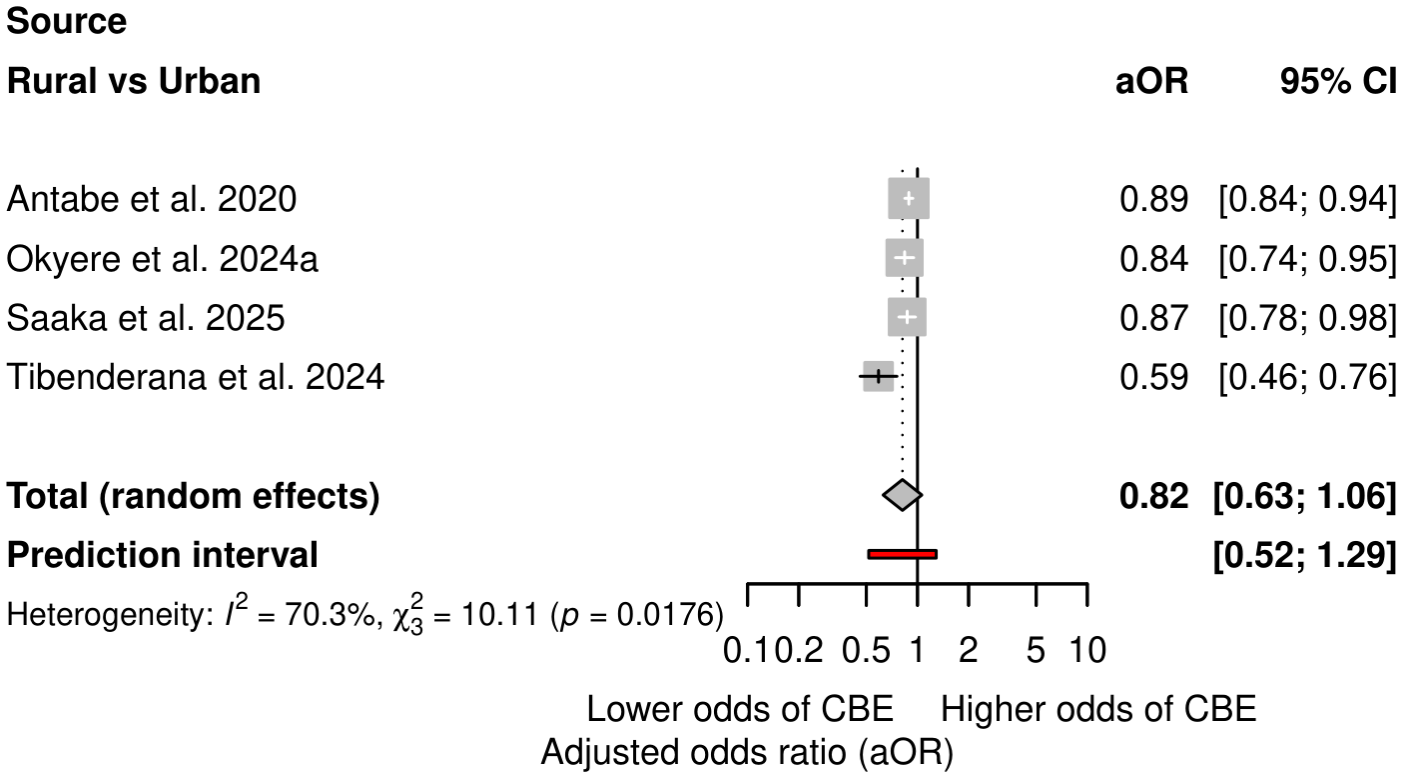
Residence (rural vs urban).

Studies (12, 15, 16, 19, 33, 41–43) found that place of residence was associated with CBE. In the independent pooled analysis, rural residence was associated with a lower, but not statistically significant, CBE uptake compared with urban residence (OR, 0.82; 95% CI, 0.63-1.06) (**Table 3**) (**Figure 9**).

Other community-level factors include community literacy (40), social norms, and attitudes toward domestic violence (3), and cultural or religious norms that may affect the acceptability of screening (45) (**Table 2**).

### Organizational and health-system factors

#### Health insurance

**Figure 10:**
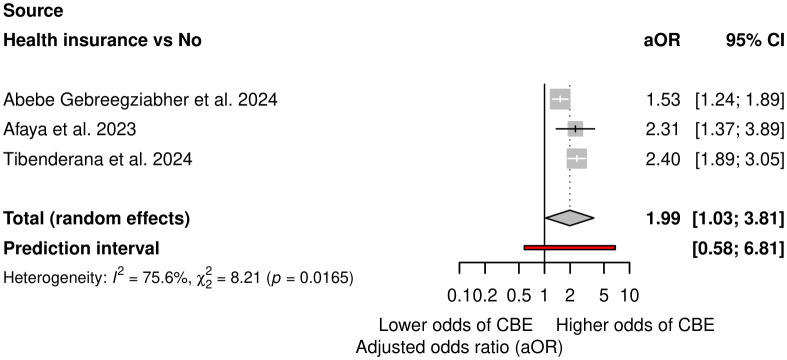
Health insurance (yes vs no).

Studies (15, 17–19, 42) found that having health insurance was associated with CBE. Health insurance coverage showed a positive pooled association with CBE (OR, 1.99; 95% CI, 1.03-3.81; 95% PI, 0.58-6.81; I² = 75.6%) (**Table 3**) (**Figure 10**).

#### Facility visits in the past 12 months

**Figure 11:**
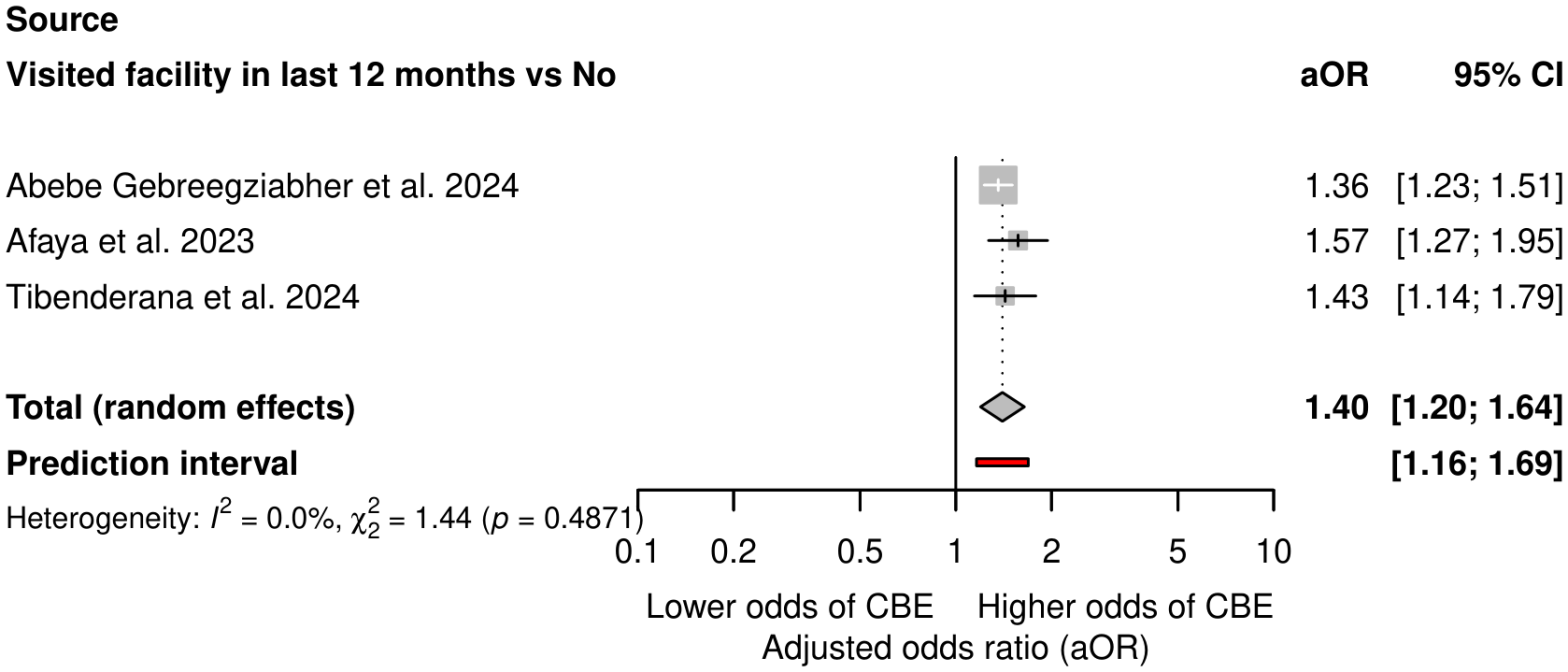
Facility visit in past 12 months (yes vs no).

Studies (11, 16–19, 42) found that having visited a health facility in the last 12 months was associated with CBE. Having visited a facility in the last 12 months was associated with higher odds of CBE (OR, 1.40; 95% CI, 1.20-1.64), with low heterogeneity (I² = 0.0%) and a prediction interval that remained above 1.0, indicating a generally consistent positive association across comparable future settings (**Table 3**) (**Figure 11**).

Additional organizational and health-system factors included perceived distance to health facilities (12, 15, 17), and awareness of locally available screening services and availability or cultural acceptability of screening services (45) (**Table 2**).

## Discussion

In our systematic review and meta-analysis, we found that CBE uptake in SSA is relatively low, with a pooled prevalence of approximately 13% and very high between-study heterogeneity (99.6%), indicating that CBE uptake varies markedly across comparable settings. Regional subgroup estimates were similarly low in East Africa (10%), Southern Africa (15%), and West Africa (14%), with no statistically significant differences among subgroups. Together, these findings suggest that limited CBE uptake is a common pattern across SSA, while the high heterogeneity points to meaningful contextual and measurement differences across countries. Although between-study heterogeneity was substantial, this likely reflects real differences in health-system capacity, screening opportunity, study populations, and contextual barriers across SSA rather than a single uniform pattern of CBE uptake. The pooled prevalence should be interpreted in relation to how CBE uptake was measured. In most included studies, CBE was assessed as a self-reported lifetime event without validation against clinical records, facility registers, or screening logs. This is important because interviewer administered health surveys in SSA may be affected by social desirability and “correct-answer” bias, particularly when respondents are asked about behaviors that are perceived as desirable or expected (46). Evidence from health-focused household surveys in four SSA countries also suggests that interviewer characteristics can influence the reporting of household and reproductive health events, with reported odds varying by about 9% to 29% for selected indicators (47). These estimates indicate that reporting differences in SSA survey data can be large enough to affect interpretation. The 13% estimate should therefore be understood as self-reported CBE uptake, not documented provider-performed CBE in routine practice. The true documented prevalence may be lower, although the available CBE evidence does not allow the magnitude of this potential inflation to be quantified. The low pooled prevalence aligns with existing evidence indicating that late-stage diagnosis is common in SSA and that deficiencies in awareness, access, and timely diagnostic pathways significantly contribute to poor outcomes (5, 48, 49). For instance, a systematic review and meta-analysis on stage at diagnosis in SSA found that late-stage presentation is prevalent and identified system-level factors, including limited early-detection programs and restricted diagnostic and treatment capacity (49).

The factors associated with CBE synthesized in our study reinforce a consistent social gradient in CBE uptake. Higher educational attainment and greater household wealth remained significantly associated with CBE uptake. These socio economic factors are plausibly explained by differences in health literacy, perceived susceptibility, and the ability to overcome financial and logistical barriers to clinic attendance, mechanisms widely noted in the SSA breast cancer early-detection literature (48, 50).

Rural residence and radio exposure showed associations in the expected direction but were not statistically significant in the pooled analysis, while internet exposure was not statistically associated with CBE. These findings suggest that information access and structural context may vary across settings. However, access to and trust in information (51) can vary across contexts due to differences in health literacy, stigma, sociocultural barriers, and structural access constraints (48). Furthermore, awareness may not transfer into action when diagnostic, referral, and treatment pathways are inadequate, because early detection without timely diagnosis and treatment is inefficient (50).

Our review also highlights health-system factors as potentially high-leverage intervention points. Health insurance was positively associated with CBE, and a facility visit in the past 12 months also showed a positive pooled association. These associations show that financial protection and recent contact with the health system are important drivers of CBE uptake, and that increasing insurance coverage while using frequent facility visits may create practical opportunities to improve CBE delivery. Evidence from a systematic review conducted in SSA found that CBE can be a resource-appropriate approach when combined with comprehensive care programs, but follow-up after screening remains a major challenge in real-world implementation (50). The WHO early diagnosis guidelines and the Global Breast Cancer Initiative framework both emphasize that early detection must be linked to timely diagnostic evaluation and access to appropriate treatment to translate into survival gains (51).

Our findings should also be interpreted in light of the evolving evidence base for CBE as a screening modality. Studies have shown that CBE can help with downstaging, but it does not consistently lower mortality (52, 53). A cluster-randomized study in Trivandrum reported a stage shift but concluded that triennial CBE screening did not result in a mortality benefit over time (52). In SSA, where organized mammography programs may be limited (48), these findings support the WHO’s emphasis on early detection systems, which include CBE as part of clinical evaluation, diagnostic confirmation, and treatment access, rather than assuming that increasing CBE alone will improve outcomes (51).

## Study strengths and limitations

Our study was preregistered in PROSPERO, and followed the PRISMA guidelines, including comprehensive database searches, independent screening and quality appraisal, and a random-effects synthesis across studies from the East, West, and Southern regions of SSA. Our study has several limitations. First, the high heterogeneity in the pooled prevalence and across many associated factors indicates substantial between-study variation in populations, recall periods, and health-system contexts. Therefore, the pooled prevalence provides a regional overview of CBE uptake but does not describe the situation in any specific SSA country. Second, CBE uptake was largely self-reported and not validated against clinical records or facility registers, potentially introducing recall and social desirability biases. Third, several included articles analyzed overlapping DHS country-survey waves; therefore, determinant meta-analyses retained only one adjusted estimate per country-survey wave for each determinant, while overlapping estimates were retained for narrative synthesis. The small number of independent estimates per determinant also limited formal assessment of publication bias or small-study effects. Fourth, although the 2021 launch of the WHO Global Breast Cancer Initiative was an important policy milestone, a pre- and post-2021 subgroup analysis was not conducted because the included studies largely measured lifetime history of CBE and did not assess country-level GBCI implementation or exposure. Fifth, because the included studies are cross-sectional, the pooled associations should be interpreted as correlational and may reflect unmeasured confounding. Sixth, we limited our search and inclusion criteria to English-language publications due to a lack of translation resources. This may have resulted in the exclusion of non-English CBE studies from francophone and lusophone SSA countries.

Implications for future research

The findings from our study point to the need for future research on CBE in SSA. First, because the current evidence used in our study is dominated by cross-sectional, largely DHS-based surveys, longitudinal and implementation studies are needed to examine whether integrating CBE into routine primary care, reproductive health, and other frontline services improves sustained uptake and timely diagnostic follow-up. Second, because the included studies relied on self-reported CBE without validation against clinical records, facility registers, or screening logs, prospective validation studies should compare survey reports with documented provider-performed examinations to quantify misclassification and strengthen coverage monitoring. Third, the socio-ecological patterning observed in this review underscores the need for context-specific qualitative and mixed-methods research to examine how sociocultural norms, gender relations, and local health-system constraints shape opportunities for provider-performed CBE.

## Conclusion

In summary, CBE uptake in SSA remains very low despite the urgent call for earlier breast cancer detection. Our findings suggest that simply expanding standalone CBE may be insufficient unless it is embedded within broader health system strengthening. Policymakers should leverage existing contact points, reduce financial barriers, and use context-appropriate communication channels to raise awareness. Also, screening efforts should be tied to functional diagnostic and treatment pathways. In resource-limited SSA settings, the early detection strategy is likely to focus on downstaging through awareness and routine clinical examination, rather than opportunistic mammography.
