# Supplementary material for: Prevalence and multilevel factors associated with clinical breast examination uptake among women in sub-Saharan Africa: a systematic review and meta-analysis
Source: Front Oncol. 2026 Jul 31;16:1856988. doi: 10.3389/fonc.2026.1856988 (PMC13472788; doi:10.3389/fonc.2026.1856988)
Supplement: Supplementary file 1 [file Table1.docx]

**Supplementary Table 1**

| **Author, year** | **Country/countries represented** | **Dataset or survey wave** | **Contribution to pooled prevalence synthesis** | **Contribution to determinant synthesis** |
| --- | --- | --- | --- | --- |
| Abebe Gebreegziabher et al. 2024 | Ghana | Ghana DHS 2022 | Ghana 2022 estimate | Pooled determinants: health insurance, facility visit, radio |
| Abubakari et al. 2025 | Ghana | Community-based survey, Kumasi, 2024 | Not included | Narrative synthesis |
| Abuhay et al. 2025 | Kenya | Kenya DHS 2022 | Kenya 2022 estimate | Pooled determinant: education |
| Addo et al. 2023 | Burkina Faso, Cote d’Ivoire, Kenya, Lesotho, Namibia | DHS: Burkina Faso 2010; Cote d’Ivoire 2011-2012; Kenya 2014; Lesotho 2014; Namibia 2013 | Burkina Faso 2010 and Cote d’Ivoire 2011-2012 estimates | Narrative synthesis |
| Afaya et al. 2023 | Lesotho | Lesotho DHS 2014 | Lesotho 2014 estimate | Pooled determinants: health insurance, facility visit |
| Ali 2025 | Kenya | Kenya DHS 2022 | Not included | Narrative synthesis |
| Antabe et al. 2020 | Kenya | Kenya DHS 2014 | Kenya 2014 estimate | Pooled determinant: residence |
| Ayebeng et al. 2025 | Burkina Faso, Cote d’Ivoire, Ghana, Kenya, Mozambique, Senegal, Tanzania | DHS: Burkina Faso 2021; Cote d’Ivoire 2021; Ghana 2022; Kenya 2022; Mozambique 2022-2023; Senegal 2023; Tanzania 2022 | Burkina Faso 2021, Cote d’Ivoire 2021, and Mozambique 2022-2023 estimates | Narrative synthesis |
| Ba et al. 2020 | Burkina Faso, Cote d’Ivoire, Kenya, Namibia | DHS: Burkina Faso 2010; Cote d’Ivoire 2011-2012; Kenya 2014; Namibia 2013 | Not included | Narrative synthesis |
| Bamusi et al. 2024 | Tanzania | Tanzania DHS 2022 | Tanzania 2022 estimate | Narrative synthesis |
| Bawuah & Edefo 2025 | Senegal | Senegal DHS 2023 | Senegal 2023 estimate | Pooled determinants: wealth, internet use, radio |
| Daniels-Donkor & Afaya 2025 | Kenya | Kenya DHS 2022 | Not included | Pooled determinant: internet use |
| Hailegebireal et al. 2024 | Burkina Faso, Cote d’Ivoire, Kenya, Lesotho, Namibia, Tanzania | DHS: Burkina Faso 2021; Cote d’Ivoire 2021; Kenya 2022; Lesotho 2014; Namibia 2013; Tanzania 2022 | Not included | Narrative synthesis |
| Kangmennaang et al. 2019 | Namibia | Namibia DHS 2013 | Namibia 2013 estimate | Narrative synthesis |
| Lemma Seifu et al. 2024 | Burkina Faso, Cote d’Ivoire, Kenya, Lesotho, Namibia, Tanzania | Multi-country DHS datasets | Not included | Narrative synthesis |
| Okyere et al. 2024a | Cote d’Ivoire | Cote d’Ivoire DHS 2021 | Not included | Pooled determinants: education, wealth, radio, residence |
| Okyere et al. 2024b | Ghana | Ghana DHS 2022 | Not included | Pooled determinant: internet use |
| Saaka et al. 2025 | Ghana | Ghana DHS 2022 | Not included | Pooled determinants: education, wealth, residence |
| Tibenderana et al. 2024 | Tanzania | Tanzania DHS 2022 | Not included | Pooled determinants: education, health insurance, facility visit, residence |

**Note.** DHS = Demographic and Health Survey. For the pooled prevalence synthesis, each included estimate represented a unique country-survey wave. For determinant meta-analyses, one adjusted estimate per country-survey wave was retained for each determinant. Studies not contributing to revised pooled determinant estimates were retained in the review and summarized narratively.
