# Supplementary material for: Prevalence and multilevel factors associated with clinical breast examination uptake among women in sub-Saharan Africa: a systematic review and meta-analysis
Source: Front Oncol. 2026 Jul 31;16:1856988. doi: 10.3389/fonc.2026.1856988 (PMC13472788; doi:10.3389/fonc.2026.1856988)
Supplement: Supplementary file 2 [file Table2.docx]

**Supplementary Table 2**

**Database search**

| **PubMed**  12/23/2025  571 results  English  1/1/2010- | (((("Early Detection of Cancer"[MeSH Terms] OR "screen*"[Text Word]) AND ("Breast Neoplasms"[MeSH Terms] OR ("breast"[Text Word] AND ("carcinoma*"[Text Word] OR "cancer*"[Text Word]))) AND ("uptake"[Text Word] OR "prevalence"[Text Word] OR "compliance"[Text Word] OR "determinants"[Text Word] OR "facilitators"[Text Word] OR "barriers"[Text Word] OR “predictors”[Text Word])) OR (("clinical"[Text Word] AND "breast"[Text Word] AND "exam*"[Text Word]) OR "CBE"[Text Word])) AND ("Algeria"[Text Word] OR "Angola"[Text Word] OR "Benin"[Text Word] OR "Botswana"[Text Word] OR "Burkina Faso"[Text Word] OR "Burundi"[Text Word] OR "Cabo Verde"[Text Word] OR "Cape Verde"[Text Word] OR "Cameroon"[Text Word] OR "Central African Republic"[Text Word] OR "Chad"[Text Word] OR "Comoros"[Text Word] OR "Congo"[Text Word] OR "Cote d'Ivoire"[Text Word] OR "Ivory Coast"[Text Word] OR "Djibouti"[Text Word] OR "Egypt"[Text Word] OR "Eritrea"[Text Word] OR "Eswatini"[Text Word] OR "Ethiopia"[Text Word] OR "Gabon"[Text Word] OR "Gambia"[Text Word] OR "Ghana"[Text Word] OR "Guinea"[Text Word] OR "Guinea-Bissau"[Text Word] OR "Kenya"[Text Word] OR "Lesotho"[Text Word] OR "Liberia"[Text Word] OR "Libya"[Text Word] OR "Madagascar"[Text Word] OR "Malawi"[Text Word] OR "Mali"[Text Word] OR "Mauritania"[Text Word] OR "Mauritius"[Text Word] OR "Morocco"[Text Word] OR "Mozambique"[Text Word] OR "Namibia"[Text Word] OR "Niger"[Text Word] OR "Nigeria"[Text Word] OR "Rwanda"[Text Word] OR "Sao Tome"[Text Word] OR "Principe"[Text Word] OR "Senegal"[Text Word] OR "Seychelles"[Text Word] OR "Sierra Leone"[Text Word] OR "Somalia"[Text Word] OR "South Africa"[Text Word] OR "Swaziland"[Text Word] OR "Sudan"[Text Word] OR "Tanzania"[Text Word] OR "Togo"[Text Word] OR "Uganda"[Text Word] OR "Zambia"[Text Word] OR "Zimbabwe"[Text Word] OR "sub saharan africa"[Text Word] OR "subsaharan africa"[Text Word] OR "SSA"[Text Word] OR "Southern Africa"[Text Word] OR "East Africa"[Text Word] OR "West Africa"[Text Word] OR "Central Africa"[Text Word] OR "Africa South of the Sahara"[MeSH Terms])) |
| --- | --- |

| **Embase**  12/23/2025  950 results  English  1/1/2010- | (('cancer screening'/exp OR screen*:ti,ab,kw) AND ('breast tumor'/exp OR (breast:ti,ab,kw AND (carcinoma*:ti,ab,kw OR cancer*:ti,ab,kw))) AND (uptake:ti,ab,kw OR prevalence:ti,ab,kw OR compliance:ti,ab,kw OR determinants:ti,ab,kw OR facilitators:ti,ab,kw OR barriers:ti,ab,kw OR predictors:ti,ab,kw) OR (clinical:ti,ab,kw AND breast:ti,ab,kw AND exam*:ti,ab,kw) OR cbe:ti,ab,kw) AND (algeria:ti,ab,kw OR angola:ti,ab,kw OR benin:ti,ab,kw OR botswana:ti,ab,kw OR 'burkina faso':ti,ab,kw OR burundi:ti,ab,kw OR 'cabo verde':ti,ab,kw OR 'cape verde' OR cameroon OR 'central african republic' OR chad OR comoros:ti,ab,kw OR congo:ti,ab,kw OR cote:ti,ab,kw OR 'ivory coast':ti,ab,kw OR djibouti:ti,ab,kw OR egypt:ti,ab,kw OR eritrea:ti,ab,kw OR eswatini:ti,ab,kw OR ethiopia:ti,ab,kw OR gabon:ti,ab,kw OR gambia:ti,ab,kw OR ghana:ti,ab,kw OR guinea:ti,ab,kw OR 'guinea bissau':ti,ab,kw OR kenya:ti,ab,kw OR lesotho:ti,ab,kw OR liberia:ti,ab,kw OR libya:ti,ab,kw OR madagascar:ti,ab,kw OR malawi:ti,ab,kw OR mali:ti,ab,kw OR mauritania:ti,ab,kw OR mauritius:ti,ab,kw OR morocco:ti,ab,kw OR mozambique:ti,ab,kw OR namibia:ti,ab,kw OR niger:ti,ab,kw OR nigeria:ti,ab,kw OR rwanda:ti,ab,kw OR 'sao tome':ti,ab,kw OR principe:ti,ab,kw OR senegal:ti,ab,kw OR seychelles:ti,ab,kw OR 'sierra leone':ti,ab,kw OR somalia:ti,ab,kw OR 'south africa':ti,ab,kw OR swaziland:ti,ab,kw OR sudan:ti,ab,kw OR tanzania:ti,ab,kw OR togo:ti,ab,kw OR uganda:ti,ab,kw OR zambia:ti,ab,kw OR zimbabwe:ti,ab,kw OR 'subsaharan africa':ti,ab,kw OR 'sub saharan africa':ti,ab,kw OR ssa:ti,ab,kw OR 'southern africa':ti,ab,kw OR 'east africa':ti,ab,kw OR 'west africa':ti,ab,kw OR 'central africa':ti,ab,kw OR 'africa south of the sahara'/exp) |
| --- | --- |

| **CINAHL**  **12/23/2025**  133 results  English  01/01/2010- | (((MH "Cancer Screening" OR XB screen* ) AND (MH "Breast Neoplasms" OR (XB breast AND (XB carcinoma* OR XB cancer* ))) AND (XB uptake OR XB prevalence OR XB compliance OR XB determinants OR XB facilitators OR XB barriers OR XB predictors ) OR ((XB clinical AND XB breast AND XB exam* ) OR XB CBE )))  AND (XB Algeria OR XB Angola OR XB Benin OR XB Botswana OR XB "Burkina Faso" OR XB Burundi OR XB "Cabo Verde" OR XB "Cape Verde" OR XB Cameroon OR XB "Central African Republic" OR XB Chad OR XB Comoros OR XB Congo OR XB "Cote d'Ivoire" OR XB "Ivory Coast" OR XB Djibouti OR XB Egypt OR XB Eritrea OR XB Eswatini OR XB Ethiopia OR XB Gabon OR XB Gambia OR XB Ghana OR XB Guinea OR XB “Guinea-Bissau” OR XB Kenya OR XB Lesotho OR XB Liberia OR XB Libya OR XB Madagascar OR XB Malawi OR XB Mali OR XB Mauritania OR XB Mauritius OR XB Morocco OR XB Mozambique OR XB Namibia OR XB Niger OR XB Nigeria OR XB Rwanda OR XB "Sao Tome" OR XB Principe OR XB Senegal OR XB Seychelles OR XB "Sierra Leone" OR XB Somalia OR XB "South Africa" OR XB Swaziland OR XB Sudan OR XB Tanzania OR XB Togo OR XB Uganda OR XB Zambia OR XB Zimbabwe OR XB "subsaharan africa" OR XB "sub saharan africa" OR XB SSA OR XB "Southern Africa" OR XB "East Africa" OR XB "West Africa" OR XB"Central Africa" OR MH "Africa South of the Sahara" OR MH “Sub-Saharan Africans” ) |
| --- | --- |

| **Africa-Wide**  **Information**  12/23/2025  250 results  English  01/01/2010- | ((( AB screen* OR TI screen* ) AND ("Breast Neoplasms" OR ((AB breast OR TI breast) AND (AB carcinoma* OR TI carcinoma* OR AB cancer* OR TI cancer* ))) AND (AB uptake Or TI uptake OR AB prevalence OR TI prevalence OR AB compliance OR TI compliance OR AB determinants OR TI determinants OR AB facilitators OR TI facilitators OR AB barriers OR TI barriers OR AB predictors OR TI predictors ) OR (((AB clinical OR TI clinical) AND ( AB breast OR TI breast) AND (AB exam* OR TI exam*) OR (AB CBE OR TI CBE) )))  AND  (AB Algeria OR TI Algeria OR AB Angola OR TI Angola OR AB Benin OR TI Benin OR AB Botswana OR TI Botswana OR AB "Burkina Faso" OR TI “Burkina Faso” OR AB Burundi OR TI Burundi OR AB "Cabo Verde" OR TI “Cabo Verde” OR AB "Cape Verde" Or TI “Cape Verde” OR AB Cameroon OR TI Cameroon OR AB "Central African Republic" OR TI “Central African Republic” OR AB Chad OR TI Chad OR AB Comoros OR TI Comoros OR AB Congo OR TI Congo OR AB "Cote d'Ivoire" OR TI “Cote d’Ivoire” OR AB "Ivory Coast" OR TI “Ivory Coast” OR AB Djibouti OR TI Djibouti OR AB Egypt OR TI Egypt OR AB Eritrea OR TI Eritrea OR AB Eswatini OR TI Eswatini OR AB Ethiopia OR TI Ethiopia OR AB Gabon OR TI Gabon OR AB Gambia OR TI Gambia OR AB Ghana OR TI Ghana OR AB Guinea OR TI Guinea OR AB “Guinea-Bissau” OR TI “Guinea-Bissau” OR AB Kenya OR TI Kenya OR AB Lesotho OR TI Lesotho OR AB Liberia OT TI Liberia OR AB Libya OR TI Libya OR AB Madagascar OR TI Madagascar OR AB Malawi OR TI Malawai OR AB Mali OR TI Mali OR AB Mauritania OR TI Mauritania OR AB Mauritius OR TI MauritusOR AB Morocco OR TI Morocco OR AB Mozambique OR TI Mozambique OR AB Namibia OR TI Namibia OR AB Niger OR TI Niger OR AB Nigeria OR TI Nigeria OR AB Rwanda OR TI Rwanda OR AB "Sao Tome" OR TI “Sao Tome” OR AB Principe OR TI Principe OR AB Senegal OR TI Senegal OR AB Seychelles OR TI Seychelles OR AB "Sierra Leone" OR TI “Sierra Leone” OR AB Somalia OR TI Somalia OR AB "South Africa" OR TI “South Africa” OR AB Swaziland OR TI Swaziland OR AB Sudan OR TI Sudan OR AB Tanzania OR TI Tanzania OR AB Togo OR TI Togo OR AB Uganda OR TI Uganda OR AB Zambia Or TI Zambia OR AB Zimbabwe OR TI Zimbabwe OR AB "subsaharan africa" OR TI “subsaharan Africa” OR AB "sub saharan africa" OR TI “sub Saharan Africa” OR AB SSA OR TI SSA OR AB "Southern Africa" OR TI “Southern Africa” OR AB "East Africa" OR TI “East Africa” OR AB "West Africa" OR TI “West Africa” OR AB "Central Africa" OR TI “Central Africa” ) |
| --- | --- |

| **Google Scholar**  12/23/2025  Limited to 1000 most relevant citations | "clinical breast examination" OR "clinical breast exam" AND (Sub-Saharan Africa OR SSA OR Africa) |
| --- | --- |

| **Web of Science**  12/23/2025  659 results  English  Indexes searched:  Science Citation Index Expanded (SCI-EXPANDED), Social Sciences Citation Index (SSCI), Arts & Humanities Citation Index, Conference Proceedings Citation Index- Science, Conference Proceedings Citation Index – Social Sciences & Humanities, Book Citation Index – Science, Book Citation Index – Social Sciences & Humanities, Emerging Sources Citation Index | TS=((((screen* ) AND ("Breast Neoplasms" OR (breast AND (carcinoma* OR cancer* ))) AND (uptake OR prevalence OR compliance OR determinants OR facilitators OR barriers OR predictors ) OR ((clinical AND breast AND exam* ) OR CBE )))) AND  (TS=((Algeria OR Angola OR Benin OR Botswana OR "Burkina Faso" OR Burundi OR "Cabo Verde" OR "Cape Verde" OR Cameroon OR "Central African Republic" OR Chad OR Comoros OR Congo OR "Cote d'Ivoire" OR "Ivory Coast" OR Djibouti OR Egypt OR Eritrea OR Eswatini OR Ethiopia OR Gabon OR Gambia OR Ghana OR Guinea OR “guinea-Bissau” OR Kenya OR Lesotho OR Liberia OR Libya OR Madagascar OR Malawi OR Mali OR Mauritania OR Mauritius OR Morocco OR Mozambique OR Namibia OR Niger OR Nigeria OR Rwanda OR "Sao Tome" OR Principe OR Senegal OR Seychelles OR "Sierra Leone" OR Somalia OR "South Africa" OR Swaziland OR Sudan OR Tanzania OR Togo OR Uganda OR Zambia OR Zimbabwe OR "sub saharan africa" OR "sub saharan africa" OR SSA OR "Southern Africa" OR "East Africa" OR "West Africa" OR "Central Africa")) |
| --- | --- |

**Supplementary Table 3**

**Websites searched in the grey literature search**

| **Name, Link, & Date Searched** | **Strategy/Comments** |
| --- | --- |
| WHO Institutional Repository for Information Sharing (IRIS)  https://iris.who.int/  16 May 2026  53 results | Searched repository using the IRIS search interface. Used the terms:  1. "clinical breast examination" AND (Africa OR "sub-Saharan Africa")  2. "clinical breast examination" AND (Ghana OR Kenya OR Tanzania OR Senegal OR "Cote d'Ivoire" OR "Ivory Coast" OR "Burkina Faso" OR Lesotho OR Namibia OR Mozambique OR Ethiopia)  3. "breast examination" AND (screening OR "early detection" OR "early diagnosis") AND (Africa OR "sub-Saharan Africa")  4. "breast cancer" AND (screening OR "early detection" OR "early diagnosis") AND (policy OR guideline OR strategy OR framework OR implementation) AND (Africa OR "sub-Saharan Africa")  5. Combined cross-check query |
| WHO Regional Office for Africa (AFRO)  https://www.afro.who.int/  16 May 2026 | 1. clinical breast examination  2. breast examination  3. "breast cancer" AND screening  4. "breast cancer" AND "early diagnosis OR early detection"  5. "breast cancer" AND guideline OR strategy |
| WHO Global Health Observatory (GHO), Indicators index  https://www.who.int/data/gho/data/indicators/indicators-index  18 May 2026  No results | 1. breast cancer  2. clinical breast examination  3. CBE  4. cancer screening  5. early detection  6. early diagnosis  7. screening coverage |
| WHO Global Breast Cancer Initiative / WHO breast cancer pages  https://www.who.int/initiatives/global-breast-cancer-initiative  18 May 2026  4 results | "clinical breast examination"; "CBE"; "breast examination"; "screening"; "early detection"; "early diagnosis"; "Africa"; and "sub-Saharan Africa". |
| African Index Medicus  https://search.bvsalud.org/aimafro/  18 May 2026  13 results | "clinical breast examination," "clinical breast exam," CBE, "breast examination," "breast exam," "breast cancer screening," "breast cancer early detection," and "breast screening." Records were screened for CBE uptake, screening practice, prevalence, barriers, and determinants. |
| National Ministry of Health / national cancer-control sources for countries represented in the review  Official country MoH/NCCP pages and official national-plan repositories (see Table X3)  18 May 2026  3 results | Targeted review of official government/national sources for countries represented in the included evidence base. Priority documents were national cancer-control plans, national cancer screening guidelines/protocols, annual health-statistics reports, and official programme documents mentioning CBE, breast examination, breast screening, or early detection. English-language national/contextual documents were retained only when relevant. No relevant English-language national CBE-specific document was retained for Lesotho or Namibia, and non-English francophone or lusophone documents were not extracted because translation services were unavailable. |
| IARC / Global Cancer Observatory  https://gco.iarc.who.int/  18 May 2026  No results | Breast exam, breast screening |

**Supplementary Table 4. National official documents government/national repositories**

| **Country** | **National/official source** | **Document** |
| --- | --- | --- |
| Kenya | National Cancer Institute Kenya / Ministry of Health | Kenya National Cancer Screening Guidelines. <https://uploads.ncikenya.go.ke/uploads/policy/National-Cancer-Screening-Guidelines.pdf> |
| Ghana | Ministry of Health / national cancer-control plan | National Strategy for Cancer Control in Ghana. [https://www.iccp-portal.org/sites/default/files/plans/Cancer%20Plan%20Ghana%20Ministry%20of%20Health.pdf](https://www.iccp-portal.org/sites/default/files/plans/Cancer%20Plan%20Ghana%20Ministry%20of%20Health) |
| Tanzania | Ministry of Health | Health Sector Strategic Plan V. [https://www.moh.go.tz/storage/app/uploads/public/672/630/145/6726301451e92438381140.pdf](https://www.moh.go.tz/storage/app/uploads/public/672/630/145/6726301451e92438381140) |
